# High fitness levels, frequent sauna bathing and risk of pneumonia in a cohort study: Are there potential implications for COVID‐19?

**DOI:** 10.1111/eci.13490

**Published:** 2021-01-22

**Authors:** Setor K. Kunutsor, Jari A. Laukkanen

**Affiliations:** ^1^ National Institute for Health Research Bristol Biomedical Research Centre University Hospitals Bristol and Weston NHS Foundation Trust and the University of Bristol Bristol UK; ^2^ Musculoskeletal Research Unit, Translational Health Sciences Bristol Medical School Learning & Research Building (Level 1), Southmead Hospital University of Bristol Bristol UK; ^3^ Institute of Public Health and Clinical Nutrition University of Eastern Finland Kuopio Finland; ^4^ Department of Medicine Institute of Clinical Medicine University of Eastern Finland Kuopio Finland; ^5^ Department of Medicine Central Finland Health Care District Hospital District Jyväskylä Finland

**Keywords:** cardiorespiratory fitness, COVID‐19, pneumonia, sauna

## Abstract

**Background:**

There is an ongoing debate on a potential protective role of habitual physical activity and passive heat therapy on the risk of COVID‐19, a respiratory infectious disease that can manifest as severe pneumonia. To explore these putative roles, we evaluated the independent and joint associations of cardiorespiratory fitness (CRF) and frequency of sauna bathing (FSB) with pneumonia risk in a prospective cohort study of 2275 men aged 42‐61 years at recruitment.

**Material and Methods:**

Objectively measured CRF and self‐reported sauna bathing habits were assessed at baseline. CRF was categorized as low and high (median cut‐offs) and FSB as low and high (defined as ≤1 and 2‐7 sessions/wk, respectively). Multivariable‐adjusted hazard ratios (HRs) with confidence intervals (CIs) were calculated for incident pneumonia.

**Results:**

During a median follow‐up of 26.6 years, 529 cases of pneumonia occurred. Comparing high vs low CRF, the multivariable‐adjusted HR (95% CIs) for pneumonia was 0.75 (0.61‐0.91). Comparing high vs low FSB, the corresponding HR was 0.81 (0.68‐0.97). Compared to men with low CRF & low FSB, the multivariable‐adjusted HRs of pneumonia for the following groups: high CRF & low FSB; low CRF & high FSB; and high CRF & high FSB were 0.88 (0.65‐1.20), 0.89 (0.71‐1.13), and 0.62 (0.48‐0.80) respectively.

**Conclusions:**

In a general male Caucasian population, a combination of high fitness levels and frequent sauna baths is associated with a substantially lowered future pneumonia risk compared with each modality alone. The implications of these findings in altering COVID‐19 disease or its severity deserve study.

## 1 INTRODUCTION

Pneumonia infection is the result of a complex inflammatory process where the lower respiratory tract suffers the invasion of an infective microorganism (viruses or bacteria). Major risk factors that predispose to pneumonia include smoking, obesity, underweight, excessive alcohol consumption and comorbid conditions such as asthma, chronic obstructive pulmonary disease, cardiovascular, kidney and liver diseases.^1^ Physical activity (PA), a potent stimulus of immune function,^2^ has been shown to be associated with a lower risk of pneumonia in a dose‐dependent manner.^3^ Cardiorespiratory fitness (CRF), as measured by maximal oxygen uptake (VO_2max_), is an index of habitual PA and considered to be the gold standard for assessing aerobic capacity.^4^ CRF has been shown to be consistently and independently associated with a reduced risk of major adverse vascular and non‐vascular outcomes in general population settings.^5^, ^6^, ^7^ In recent prospective epidemiological evaluations, we showed elevated CRF to be associated with reduced risk of pneumonia and other respiratory diseases.^8^, ^9^ Sauna bathing, a passive heat therapy commonly undertaken in Finland for the purposes of pleasure and relaxation, has been linked to several health benefits^10^, ^11^, ^12^, ^13^ including a reduced risk of respiratory tract diseases such as pneumonia.^14^, ^15^ There is evidence to suggest that CRF and sauna bathing may exert a synergistic effect on health outcomes. In recent evaluations of the joint impact of CRF and frequency of sauna bathing (FSB) on the risk of cardiovascular outcomes and all‐cause mortality, we demonstrated that high CRF and frequent sauna bathing confer stronger long‐term protection on these outcomes compared with each exposure alone.^16^, ^17^

Given the overall evidence, we hypothesized that a combination of high CRF and frequent sauna bathing might have substantial benefit for pneumonia prevention, compared with each modality alone. Using a population‐based prospective cohort of 2275 Caucasian men, we evaluated the joint effects of CRF and FSB on the risk of incident pneumonia. To enable direct comparisons between the two exposures, we initially evaluated the separate associations of CRF and FSB with the risk of pneumonia in the same participants.

## 2 METHODS

Reporting of the study conformed to broad EQUATOR guidelines^18^ and was conducted according to STROBE (STrengthening the Reporting of OBservational studies in Epidemiology) guidelines for reporting observational studies in epidemiology (Supplementary Material). Participants in the current analysis were part of the Kuopio Ischemic Heart Disease (KIHD) risk factor study, an ongoing population‐based prospective cohort study comprising a representative sample of middle‐aged men aged 42‐61 years with baseline examinations carried out between March 1984 and December 1989.^19^ The Research Ethics Committee of the University of Eastern Finland approved the study protocol, and written informed consent was obtained from all participants. CRF, as measured by maximal oxygen uptake (VO_2max_), was assessed using a respiratory gas exchange analyzer (Medical Graphics, MCG) during cycle ergometer exercise testing.^20^ Baseline sauna habits per week were assessed by a self‐administered questionnaire.^12^ Pneumonia cases that occurred from study entry to 2018 were included, and these were collected by data linkage to the National Hospital Discharge Register and a comprehensive review of all available records of hospitals and healthcare centres. The diagnoses of pneumonia cases were made by qualified physicians based on the International Classification of Diseases codes used in clinical practice.^8^ Hazard ratios (HRs) with 95% confidence intervals (CIs) for pneumonia were calculated using Cox proportional hazard models. To maintain consistency with previous reports,^16^, ^17^ CRF was categorized into low and high CRF based on median cut‐offs of CRF, whereas FSB was categorized into low and high FSB (defined as ≤1 and 2‐7 sauna sessions per week, respectively). The combined association of CRF and FSB with pneumonia risk was based on the following four possible combinations: low CRF & low FSB; high CRF & low FSB; low CRF & high FSB; and high CRF & high FSB. All statistical analyses were conducted using Stata version MP 16 (Stata Corp).

## 3 RESULTS

The mean (standard deviation, SD) age of study participants at baseline was 53 (5) years. The mean (SD) of CRF and median (interquartile range, IQR) FSB were 30.3 (8.0) ml/kg/min and 2 (1‐2) sessions per week, respectively (Table 1). During a median (IQR) follow‐up of 26.6 (17.4‐31.0) years, a total of 529 incident cases of pneumonia were recorded (annual rate 9.89/1000 person‐years at risk; 95% CI 9.08‐10.77). Compared to men with low CRF, high CRF was associated with a decreased risk of pneumonia following adjustment for potential confounders (age, body mass index (BMI), smoking status, systolic blood pressure (SBP), history of type 2 diabetes, history of coronary heart disease, history of asthma, history of chronic bronchitis, history of tuberculosis, alcohol consumption, socioeconomic status (SES), physical activity and high sensitivity C‐reactive protein (hsCRP) 0.74 (95% CI: 0.61‐0.90) and this remained unchanged on further adjustment for FSB (Table 2). On adjustment for potential confounders as above, high FSB was associated with a decreased risk of pneumonia compared with low FSB 0.79 (95% CI: 0.66‐0.94), which remained similar on additional adjustment for CRF (Table 2).

**TABLE 1:** Baseline characteristics of study participants

Characteristics	Mean (SD) or median (IQR) or n (%)
Cardiorespiratory fitness (ml/kg/min)	30.3 (8.0)
Frequency of sauna bathing (sessions/wk)	2 (1‐2)
Questionnaire/Prevalent conditions	
Age (y)	53 (5)
Alcohol consumption (g/wk)	32.0 (6.4‐92.5)
History of type 2 diabetes	79 (3.5)
Current smoking	714 (31.4)
History of CHD	538 (23.7)
History of asthma	76 (3.3)
History of chronic bronchitis	164 (7.2)
History of tuberculosis	86 (3.8)
Physical measurements	
BMI (kg/m^2^)	26.9 (3.5)
SBP (mmHg)	134 (17)
DBP (mmHg)	89 (10)
Physical activity (KJ/d)	1208 (626‐1991)
Socio‐economic status	8.42 (4.24)
Lipid markers	
Total cholesterol (mmol/L)	5.91 (1.07)
HDL‐C (mmol/L)	1.29 (0.30)
Metabolic and inflammatory markers	
Fasting plasma glucose (mmol/L)	5.33 (1.19)
C‐reactive protein (mg/L)	1.24 (0.69‐2.37)

Abbreviations: BMI, body mass index; CHD, coronary heart disease; DBP, diastolic blood pressure; GFR, glomerular filtration rate; HDL‐C, high‐density lipoprotein cholesterol; SBP, systolic blood pressure; SD, standard deviation.

**TABLE 2:** Associations of cardiorespiratory fitness and frequency of sauna bathing with risk of pneumonia

Exposure categories	Events/Total	Model 1		Model 2		Model 3	
		HR (95% CI)	*P‐*value	HR (95% CI)	*P‐*value	HR (95% CI)	*P‐*value
CRF (ml/kg/min)							
Low CRF	302/1138	ref		ref		ref	
High CRF	227/1137	0.61 (0.51‐0.73)	<.001	0.74 (0.61‐0.90)	.002	0.75 (0.61‐0.91)	.003
Frequency of sauna bathing (sessions/wk)							
Low FSB	196/800	ref		ref		ref	
High FSB	333/1475	0.72 (0.60‐0.86)	<.001	0.79 (0.66‐0.94)	.01	0.81 (0.68‐0.97)	.02
CRF (ml/kg/min) and frequency of sauna bathing (sessions/wk) combination							
Low CRF & Low FSB	116/448	ref		ref		NA	
High CRF & Low FSB	80/352	0.71 (0.54‐0.95)	.02	0.88 (0.65‐1.20)	.42	NA	
Low CRF & High FSB	186/690	0.81 (0.65‐1.03)	.08	0.89 (0.71‐1.13)	.35	NA	
High CRF & High FSB	147/785	0.48 (0.37‐0.61)	<.001	0.62 (0.48‐0.80)	<.001	NA	

Note

Model 1: Adjusted for age.

Model 2: Model 1 plus body mass index, smoking status, systolic blood pressure, history of type 2 diabetes, history of coronary heart disease, history of asthma, history of chronic bronchitis, history of tuberculosis, alcohol consumption, socioeconomic status, physical activity and high sensitivity C‐reactive protein.

Model 3: Model 2 plus FSB for CRF and CRF for FSB.

Abbreviations: CI, confidence interval; CRF, cardiorespiratory fitness; cut‐off for CRF was based on the median; FSB, frequency of sauna bathing; HR, hazard ratio; NA, not applicable; ref, reference.

**FIGURE 1:**
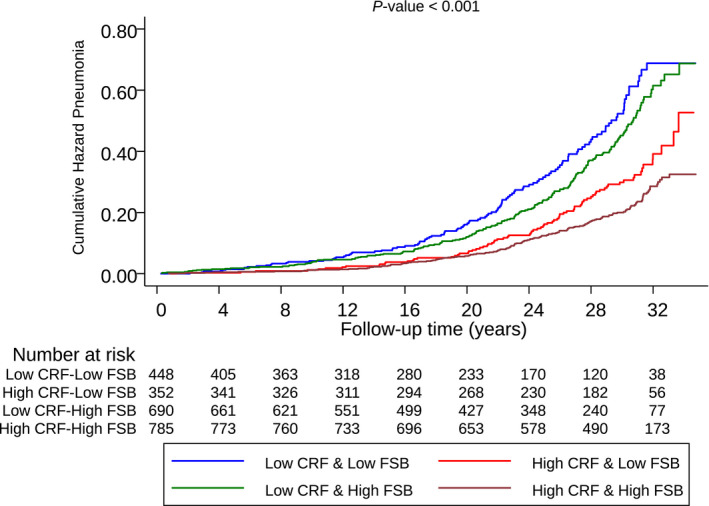
Cumulative Kaplan‐Meier curves for pneumonia during follow‐up according to joint categories of CRF and FSB. CRF, cardiorespiratory fitness; FSB, frequency of sauna bathing

In the evaluation of joint associations of CRF and FSB with the risk of pneumonia, cumulative hazard curves showed a reduced risk of pneumonia among participants with high CRF & high FSB compared with other groups (*P*‐value for log‐rank test <.001 for all; Figure 1). Compared to men with low CRF & low FSB, the multivariable‐adjusted HRs (95% CIs) of pneumonia for the following groups: high CRF & low FSB; low CRF & high FSB; and high CRF & high FSB were 0.88 (0.65‐1.20), 0.89 (0.71‐1.13) and 0.62 (0.48‐0.80), respectively.

## 4 DISCUSSION

In a general population of Finnish men, we have shown that increased baseline levels of CRF and FSB are each associated with a reduced risk of future pneumonia. These associations were independent of several established and emerging risk factors as well as each exposure. The risk of pneumonia was substantially reduced for the combined exposure of high CRF and high FSB compared with high levels of each modality alone. There was no evidence of associations for the combinations of high CRF & low FSB and low CRF & high FSB, which suggests that one exposure is not a stronger risk indicator than the other and vice versa, but both have a synergistic effect on the outcome. These findings are consistent with previous findings on the joint associations of CRF and FSB on adverse vascular outcomes as well as all‐cause mortality.^16^, ^17^ There have also been reports of substantial beneficial changes in cardiovascular function when PA is combined with sauna exposure.^21^, ^22^, ^23^

Increasing levels of PA and exercise training, particularly aerobic activity, generally confers good CRF,^24^, ^25^ which has consistently been shown to be strongly protective of vascular and non‐vascular disease as well as mortality.^26^, ^27^ PA has also been regarded as a potential immune function adjuvant to reduce the risk of communicable diseases caused by bacterial and viral infections.^2^ There is mounting epidemiological evidence on a dose‐response relationship between PA and a reduction in the incidence, duration or severity of self‐reported upper respiratory tract infections.^2^ The potential mechanisms of action underlying the protective effect of PA (as measured by CRF) include stimulation of the antipathogen activity of immune system macrophages and key immune system cells in the blood as well as suppressing inflammation in the lungs.^2^ Regular sauna bathing has traditionally been used in Finland as a method of “hardening,” which means enhancing the body's resistance. Frequent sauna sessions reduce the risk of respiratory tract infections, such as common colds and pneumonia caused by viral and bacterial infections via (a) direct inhibition of pathogens; (b) boosting both the innate and adaptive arms of the immune system; (c) dampening of inflammatory responses; and (d) by having direct effects on lung tissue which include improvement in lung function and reduction in pulmonary congestion.^28^, ^29^, ^30^ Evidence suggests the adaptive responses produced by an ordinary sauna bath corresponds to that produced by moderate or high‐intensity PA.^31^

The evidence of similar mechanistic pathways underlying the associations of CRF and sauna exposure on the risk of pneumonia appears to confirm their synergistic effects on the risk of pneumonia. High fitness levels and frequent sauna baths confer more protection for pneumonia compared with each alone. Coronavirus disease‐2019 (COVID‐19) is a novel respiratory infectious disease caused by severe acute respiratory syndrome coronavirus 2 (SARS CoV‐2). The majority of patients with COVID‐19 exhibit mild symptoms such as fever, cough and myalgia, with a few progressing to severe pneumonia, extrapulmonary manifestations and death.^32^, ^33^, ^34^, ^35^ Severe disease in COVID‐19 has been reported to be induced by a “cytokine storm syndrome” characterized by markedly elevated levels of inflammatory cytokines.^36^ Respiratory conditions such as pneumonia and COVID‐19 share common risk factors such as smoking, obesity, excessive alcohol consumption and comorbidities.^37^, ^38^ The current findings may have implications for the prevention of COVID‐19. There is a wealth of evidence on the roles of regular PA and passive heat therapy as potent immune function stimuli, their ability to provide protection from viral infections and reduce inflammation. There is an ongoing debate that high levels of CRF levels may have protective effects on SARS CoV‐2 infection by attenuating the “cytokine storm syndrome.”^39^ Furthermore, given that SARS CoV‐2 is highly sensitive to heat,^40^ there are suggestions that passive heat therapies such as Finnish saunas could be used to ward off COVID‐19 or prevent severe disease.^30^ Combining these two strategies may effectively reduce the risk of COVID‐19 or its severity, especially in the at‐risk groups like older adults and those with comorbidities. Randomized clinical trials are needed to confirm the robust effectiveness of the combination of PA and passive heat therapy in altering infection risk or prognosis.

Several strengths of this evaluation deserve mention and include the novelty, well‐characterized sample of men who were representative of the general Finnish middle‐aged male population; the objective assessment of CRF using respiratory gas analyses; the long‐term follow‐up of the cohort; and adjustment for a comprehensive list of potential confounders. The limitations included the use of self‐reported questionnaires in assessing sauna bathing frequency, which may have introduced the possibility of misclassification bias; inability to generalize the findings to women and other populations; possibility of biases such as reverse causation and residual confounding. Though our assessments of exposures were based on baseline values, potentially presenting the risk of regression dilution bias, our reproducibility studies of sauna bathing habits indicate that sauna bathing habits may be fairly consistent within Finnish individuals over several years.^41^ Finally, there may also be biases due to lack of data on specific types of pneumonia and the possibility of underestimation of pneumonia incidence due to the exclusion of undiagnosed pneumonia or cases that were not treated at a health facility (eg treated at home). However, this will be minimal considering the age group of the cohort; any suspected case of pneumonia will report to a healthcare setting rather than resort to self‐medication, which is not encouraged.

## 5 CONCLUSION

In a general Caucasian male population, a combination of high fitness levels and frequent sauna baths is associated with a substantially lowered risk of future pneumonia compared with each modality alone. The implications of these findings in altering SARS CoV‐2 infection or its severity deserve study.

## CONFLICT OF INTEREST

No potential conflict of interest was reported by the authors.

## AUTHOR CONTRIBUTIONS

SKK contributed to study design, data analysis and interpretation, drafting manuscript, and revising manuscript content and approving the final version of the manuscript; JAL contributed to study design and conduct, responsibility for the patients and data collection, and revising manuscript content and approving the final version of the manuscript.

## Supporting information

Supplementary Material
